# BFT-IoMT: A Blockchain-Based Trust Mechanism to Mitigate Sybil Attack Using Fuzzy Logic in the Internet of Medical Things

**DOI:** 10.3390/s23094265

**Published:** 2023-04-25

**Authors:** Shayan E Ali, Noshina Tariq, Farrukh Aslam Khan, Muhammad Ashraf, Wadood Abdul, Kashif Saleem

**Affiliations:** 1 Department of Computer Sciences, Shaheed Zulfikar Ali Bhutto Institute of Science and Technology, Islamabad 44000, Pakistan; shayaneali222@gmail.com; 2 Department of Avionics Engineering, Air University, Islamabad 44000, Pakistan; noshina.tariq@mail.au.edu.pk (N.T.); muhammad.ashraf@mail.au.edu.pk (M.A.); 3 Center of Excellence in Information Assurance, King Saud University, Riyadh 11653, Saudi Arabia; ksaleem@ksu.edu.sa; 4 Department of Computer Engineering, College of Computer and Information Sciences, King Saud University, Riyadh 11543, Saudi Arabia; aabdulwaheed@ksu.edu.sa

**Keywords:** trust, Internet of Things, Sybil attack, low power and lossy networks, blockchain

## Abstract

Numerous sensitive applications, such as healthcare and medical services, need reliable transmission as a prerequisite for the success of the new age of communications technology. Unfortunately, these systems are highly vulnerable to attacks like Sybil, where many false nodes are created and spread with deceitful intentions. Therefore, these false nodes must be instantly identified and isolated from the network due to security concerns and the sensitivity of data utilized in healthcare applications. Especially for life-threatening diseases like COVID-19, it is crucial to have devices connected to the Internet of Medical Things (IoMT) that can be believed to respond with high reliability and accuracy. Thus, trust-based security offers a safe environment for IoMT applications. This study proposes a blockchain-based fuzzy trust management framework (BFT-IoMT) to detect and isolate Sybil nodes in IoMT networks. The results demonstrate that the proposed BFT-IoMT framework is 25.43% and 12.64%, 12.54% and 6.65%, 37.85% and 19.08%, 17.40% and 8.72%, and 13.04% and 5.05% more efficient and effective in terms of energy consumption, attack detection, trust computation reliability, packet delivery ratio, and throughput, respectively, as compared to the other state-of-the-art frameworks available in the literature.

## 1. Introduction

The Internet of Things (IoT) consists of physical objects, such as devices, cars, structures, and objects equipped with hardware, software, sensors, and networking capabilities to collect and share data. It allows objects to be tracked and managed centrally through current device systems, allowing easier convergence of the natural world into computer-based environments. Each object is uniquely recognizable; thanks to its embedded processing system and communication over the Internet [1,2]. Customer Facing Devices (CFD) (e.g., establishing interactive interfaces within physical locations) are also included in the IoT. Specific devices are designed for businesses to allow communication among them. For instance, IoT may also represent Radio Frequency Identification (RFID) tags that businesses use to track inventory in shops and sensor systems that detect energy in homes. Furthermore, IoT devices will dominate desktop and laptop computers, even if these devices are frequently used to communicate with other “things” [3]. It has also revolutionized healthcare systems like the Internet of Medical Things (IoMT), where patients use wearable IoT devices/sensors for their well-being. IoMT is a collection of medical equipment, devices, vehicles, and interconnected networks that provide dynamic communication between patients and medical professionals.

Conventionally, IoMT utilizes a healthcare cloud to store and disseminate massive data for high-performance healthcare networks. These devices gather data from clinical units and transmit it to the cloud for assessment. Numerous weak points may be exploited to compromise IoMTs, such as wireless connection assaults [4]. Furthermore, deploying new technologies on IoMT increases the danger of exposure to new hazards, which is essential, especially in the case of life-threatening diseases, such as COVID-19. IoT devices, including IoMT devices, cannot counteract privacy and security issues, even though these are the healthcare system prerequisites [5]. This problem is mainly caused due to a variety of tiny devices used in IoMT that introduce new kinds of assaults. Moreover, it renders these systems’ present security procedures ineffective. Healthcare systems have many layers, including the sensor, networking, and cloud layers. The networking layer is accountable for IoMT devices’ connectivity, making it a potential entry point for Sybil attacks on such networks [6]. A Sybil attack is an attack in which a single node is used to have multiple active fake identities simultaneously within a network, and these fake identities are used to have a disproportionately large influence. Sybil attacks may have serious consequences, including transmitting purposely faked data to a healthcare system utilizing a compromised IoMT. Furthermore, the data may be routed using different malicious nodes supplied by a single rogue node [7]. With the expansion of the IoT, every device is now connected to the Internet, and therefore, has the potential of introducing security concerns, such as confidentiality issues and dependability constraints.

Illegal access may extract cryptographic keys and steal the sensitive information of any individual, for instance. The question of how different things or entities might have faith in one another is becoming increasingly important in communication networks. Thus, establishing trust is the most significant issue in IoT networks [8]. The trust management system in IoT and IoMT networks may be broken down into two distinct classes: centralized and distributed trust assessments. The technique for handling distributed trust focuses on a user or client level, which means that every device in a communication system examines the trustworthiness of the devices located in its immediate vicinity. This system has several flaws, including high usage of computing resources, delays, and energy consumption. IoT networks are nevertheless essential to data collection, stocking, processing, and sharing, ignoring the distributed constitution of these systems, which may also result in specific protection and privacy violations. Distributed and dispersed implementations often require minimum delays in critical applications such as real-time data analytics on the IoMT. As IoMT systems are developed to upgrade the effectiveness of health treatment supplied to patients, their safety directly impacts patient safety. Unfortunately, these systems are susceptible to several security flaws and vulnerabilities, such as the Sybil attack. A Sybil attack aims at the IoMT system and compromises the safety of the patients. For example, the Sybil attacker may fabricate, delay or send incorrect data for poor assessments, increasing a patient’s safety risks by delivering substandard or no medical treatment. Therefore, ensuring the integrity of IoMT systems is mandatory. In addition, patient’s privacy and confidentiality must also be protected. In this context, IoT implementations present a significant problem in terms of stability, energy usage, network congestion, and latency, where latency will escalate as the number of connected devices increases substantially.

In addition, centrally controlled security frameworks in more clustered and standard sensor network implementations may not guarantee scalability. Therefore, a distributed architecture will inevitably handle a decentralized but safe mechanism. The Sybil attack, which allows an attacker to exploit framework assets and control system performance, has become a threat to open-access distributed frameworks and online social networks in the IoT. Even though, in some frameworks, a small number of Sybil characters can prevent the attacker from exploiting the system, the attacker’s goal is to maximize the number of Sybil identities [9] in the overlay network (e.g., a peer-to-peer network). Therefore, this paper proposes a distributed and energy-efficient blockchain-based fuzzy trust management framework (BFT-IoMT) to overcome the current problems in trust assessment for Sybil attacks. BFT-IoMT aims to accurately distinguish Sybil characters and prevent them from misusing system resources. The proposed framework utilizes fuzzy logic and blockchain to improve the efficacy and competence of the IoMT network’s communication system in a decentralized fashion. Trust is a complex quality of an entity/device in IoMT environments where every device depends on another device throughout the multi-hop communication. In the IoMT network, users may interact with a single entity at the front end, linked to several things at the back end for providing services. Therefore, ensuring that the linked nodes are reliable and that the security mechanism is not draining the device-level resources, is essential. It also improves the privacy and safety of the devices used for interaction or communication inside the IoMT ecosystem. This study aims to establish and manage trust across various nodes while facilitating human-free communication. Nodes in the system benefit from a trust mechanism that ensures their dependability, privacy, and integrity. Consequently, these nodes can safely exchange data. The potential contributions of the proposed study are listed below.

1. A distributed energy-efficient blockchain-based architecture is proposed using a fog layer to minimize the scalability problems in centralized trust-based security structures in IoMT.
2. A cluster-based trust evaluation mechanism for detecting and isolating Sybil nodes is proposed.
3. A fog-enabled trust framework is proposed to maximize the network throughput and minimize the network latency, energy consumption, and overhead communication for improved IoMT network life.
4. Fuzzy logic is used to improve both the computing power and efficiency of the decentralized trust management system.

The rest of the paper is organized as follows: The related work is presented in Section 2. The proposed framework is detailed in Section 3. Section 4 accentuates the evaluation setup and experimentation details. The conclusion and future work are given in Section 5.

## 2. Related Work

Sybil attacks on IoMT systems have been the subject of a few studies over the past few years. The Sybil assault, for example, was addressed earlier in the research conducted in [10] using the physical pressure of moving automobiles. Sybil attacks and other security vulnerabilities to the IoT and medical monitoring devices are discussed in [11]. To mitigate Sybil attacks, an encrypted eHealth system for IoT devices has been proposed in [12]. Authors in [13] presented a trusted paradigm based on time-bound group signatures to protect communication among nodes. Almogren et al. [5] proposed a trust management system using fuzzy logic for IoMT-based health infrastructures. It is an intelligent approach for identifying Sybil or unreliable system nodes. The mechanism enables IoMT systems to gather reliable and accurate data from their surrounding nodes while ignoring the Sybil ones. A fuzzy filter and fuzzy logic processing determine the nodes’ trustworthiness. In [14], a trustworthy IoT environment for strong recognition and authentication of IoT devices is proposed based on blockchain applications. Authors are primarily interested in creating a safe virtual area called a trust bubble, which devices can trust. In this mechanism, the underlying network is divided into zones. Each zone has a master computer that uses community ID, object ID, public passwords, and a signature for its followers (a combination of three parameters). Clever contracts check the master’s object ID and community ID. A confidence bubble is generated when the evidence is determined to be true.

Tariq et al. [15] proposed a blockchain-based multi-mobile code-driven trust management framework. The proposed framework mitigated blackhole and greyhole attacks. It also improved network lifetime and performance by shipping trust-related calculations over the fog layer. Another trust-based security mechanism is proposed in [16]. The proposed work used blockchain technologies to build confidence in IoT-based applications. The authors showed that blockchain is useful for data storage to ensure end-to-end trust for the IoT framework. In [17], a service-oriented TM model is presented based on the blockchain using the responsibility chain principle. The proposed model creates TERMS for use by the service provider, which requires the user to comply with the created TERMS. Another blockchain-based reputation framework is proposed in [18], which integrates blockchain for access control between IoT devices. Their strategy considers the complex management of access privileges dependent on attribute control policies. Establishing three forms of intelligent contracts is the automatic implementation of the policies. These contracts automate the validation of attributes, the confidence calculation, and the validation of policies for entry.

Liu et al. [19] proposed a blockchain-based semi-centralized trust management framework for IoT systems. Using indirect and direct trust data, they offered a computational trust framework in which the trust value of dynamic malevolent devices was computed using a proposed decay function. It used the credibility of recommendations and a set of configurable weights. The proposed framework was assessed using simulation-based experiments in various contexts and compared their framework with two traditional models. The findings of the experiments showed that the framework successfully distinguished malicious and normal devices and lessened malicious activities. Rakesh et al. [20] proposed a protocol, BlockTrust-RPL, which is a distributed blockchain-based authentication and trust validation mechanism designed for secure objective function formulation in RPL-based IoT networks. The paper’s contribution is using blockchain technology to enable distributed trust and authentication mechanisms, a promising approach to address security challenges in RPL-based IoT networks. However, the paper lacks a comprehensive comparison with other approaches, such as centralized or hybrid trust and authentication mechanisms. Furthermore, while the paper presents simulation results, there is a lack of empirical evaluation of the proposed protocol in real-world scenarios. Moreover, the paper does not provide a thorough evaluation of the scalability of the proposed protocol, which is a critical consideration for IoT networks that involve a large number of devices. Farooq et al. [21] presented a multi-agent system based trust mechanism. It employs a multi-agent system that monitors node behaviour and allocates trust values based on their actions. The framework is designed to detect and prevent attacks like selective forwarding, Sybil, and sinkhole. They demonstrate that the proposed framework can effectively mitigate attacks while maintaining network performance and minimizing communication latency.

Malik et al. [22] presented the ’ChainTrust’ concept to control IoT supply chain trust and integrity by using blockchain. To build trust among the different goods and supply chain commodities, it is divided into a three-tier system architecture: the data, an application, and a blockchain layer. The trust and credibility module at the blockchain level evaluates the consistency of different goods and trusts in participating organizations by making observations from the data layer. It is an automated procedure based on intelligent contracts, using blockchain for each transaction. It uses an Access Control List (ACL) in the blockchain layer to ensure the fulfillment of the rule during a read-and-write data operation on the blockchain. Based on predefined circumstances, smart contracts often issue alert incidents. In addition, Asif et al. [23] proposed a blockchain-based security mechanism for granting authorized user access to smart city resources safely and reliably. The said technique is built on the Object Security Architecture (OSCAR) for the Internet of Things object security model and the Authentication and Authorization for Constrained Environments (ACE) framework-based authorization blockchain. OSCAR utilizes a public ledger to set up multicast groups for approved users, much as the blockchain does with its authorization process; however, OSCAR also allows for a high degree of customization and transparency among users. In addition, a meteor-based application is also built to serve as a welcoming interface for the smart city’s disparate technology. It allows users to communicate with and manage smart municipal infrastructure, including electric meters, traffic signals, and smart cameras. However, there is an increase in the average hand-shaking time with an increase in the number of clients.

## 3. Proposed Framework

Blockchain looks suitable for technologies such as crypto-currency and anonymity to tackle the security problems of today’s ICT landscape. This paper reflects on how and why power saving, device security, latency improvements, and sustainability are positively affected by the blockchain in an IoMT network. The basic principle of blockchain technology offers the basis for collaboration between unfamiliar and untrustworthy organizations and supports the hierarchical design of the IoMT [24]. It is because the standard of modern cloud computing architectures [25] does not require a central security and authentication authority. Blockchain primarily provides distributed trust data storage and records measured trust values on the respective fog nodes. The proposed mechanism supports IoMT’s distributed and modular existence by adopting the blockchain’s controlled, decentralized, and immutable architecture.

### 3.1. The Proposed Architecture

The proposed architecture has two layers, an IoMT or infrastructure layer and a fog layer. Both these layers consist of sub-modules. Following are the elaborated details and functionality of each layer and module.

**Figure 1:**
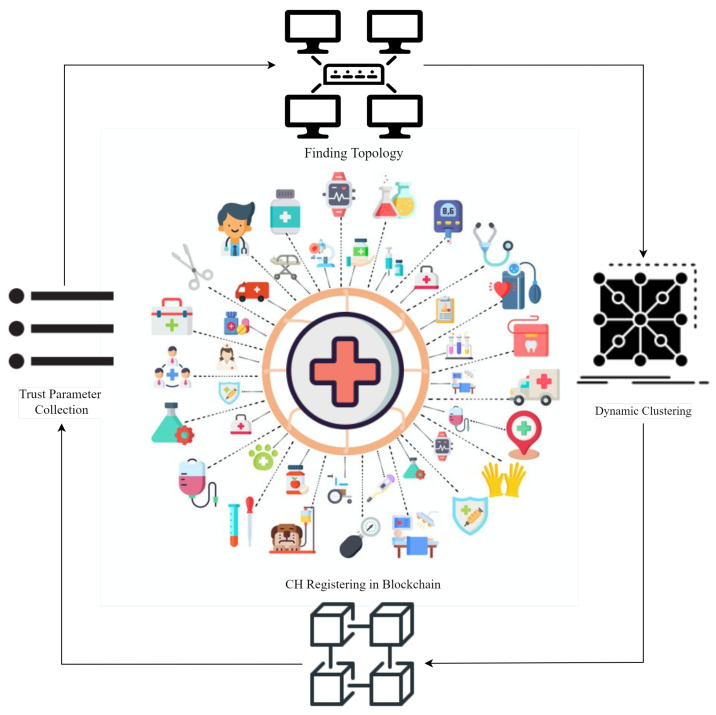
Functions of IoMT Layer.

**Figure 2:**
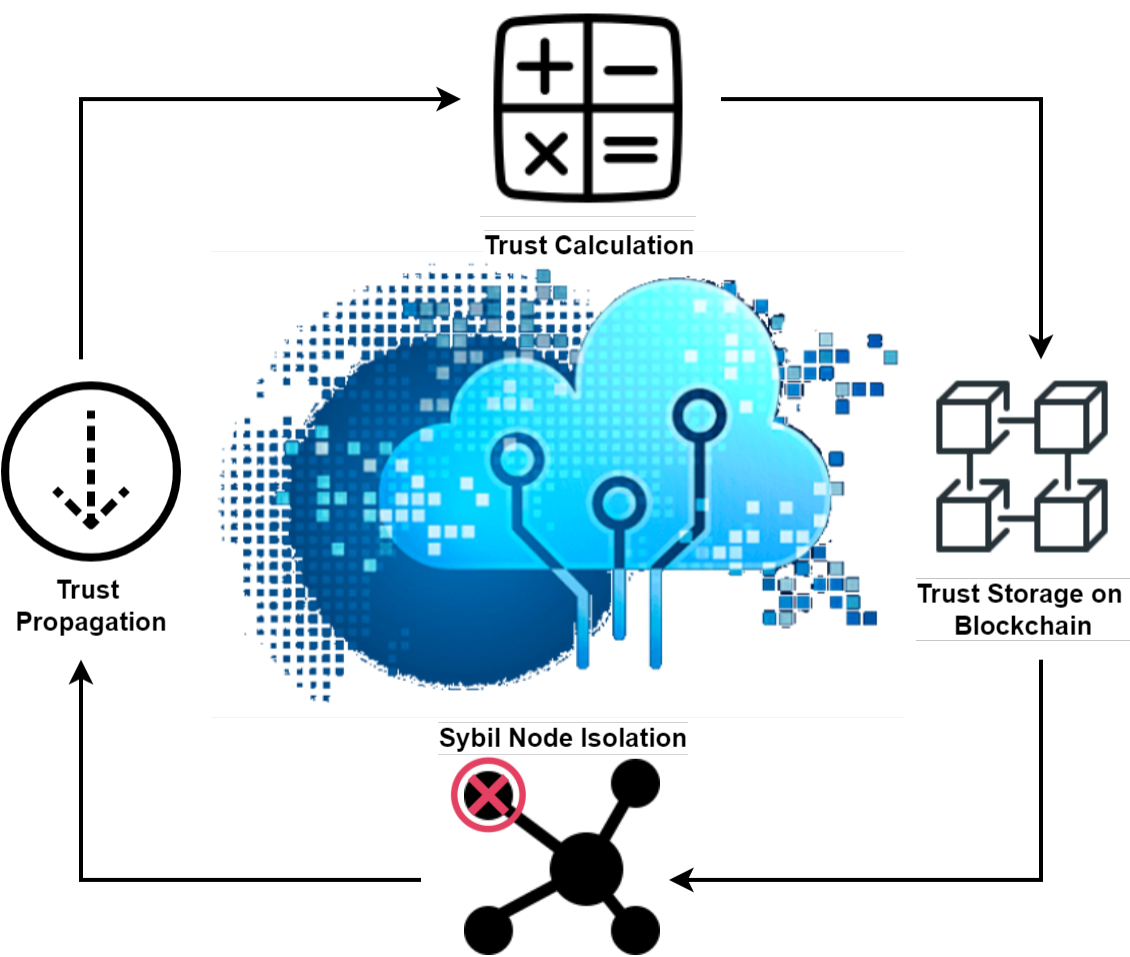
Functionalities of the Fog Layer.

1. IoMT/Infrastructure Layer
  This layer includes IoMT nodes as Sensor Nodes (SNs) used for sensing, acting, and interacting in diverse contexts (later known as healthcare networks and smart homes). Different systems’ SNs or IoMT nodes often need to connect to share data to complete the mission. Further contact can be halted if either device/node becomes malicious, launching a Sybil attack. The attack situations negatively impact the performance and lifespan of IoMT networks. The victim and intermediate nodes can generate message and energy overheads when they re-send (in the event of a multi-hop packet forwarding) the missing packets. The environment also has to be reliable and credible to ensure safe communications. An IoMT node confidence must also be considered before data gathering and intermediate data communication. Instead of, for example, changing it, the receiver can use the data correctly. Single-point loss is avoided with the proposed blockchain-enabled trust framework (as done in centralized confidence mechanisms). There is a decentralized agreement on the addition and assessment of trust and the scalability to reach IoT’s decentralized but expanding infrastructures. This layer has different functionalities, such as discovering topology, arranging logical clusters of IoMT nodes, registering Cluster Heads (CHs) in the blockchain, and gathering trust parameters, as shown in Figure 1.
  (a) Topology Discovery: It discovers the total number of active IoMT devices in each cluster. The gateway accomplishes this by providing information about all connected devices, such as their IP address, *MAC* address, and kind (sensor or actuator). These details are forwarded to the topology lookup module.
  (b) Dynamic Clustering: It uses a clustering technique to carry out dynamic clustering of nodes for trust parameter gathering.
  (c) Node and Cluster Head Registration: The list of nodes is maintained in the blockchain.
  (d) Trust Parameter Gathering: The registered IoT devices that wish to communicate with each other for data exchange have specific trust values. CHs will gather the values of predefined parameters required to calculate trust.
2. Fog Layer
  The fog layer includes blockchain-based fog nodes that help confidence assessment in creating and sustaining a safe and stable environment using various modules. They provide additional security services, such as networking, trust processing and analysis, trust storage for distributed IoMT nodes, and security [26] (i.e., encryption, an innate feature of blockchain). The fog layer takes details from the IoMT layer, detects all connected nodes, makes clusters, calculates trust, isolates malicious entities, and updates the blockchain, as shown in Figure 2. For all the nodes involved, blockchain offers an immutable record of trust values that may be accessed at any time. It ensures that trust values are correct in environments without trust, and data demands protection from being changed or faked. The fog layer contains the following modules for trust calculation:
  (a) Topology Lookup Module: With the help of the IoMT layer, it detects the cumulative number of IoMT nodes in the network. The gateway contains all linked device information, such as *MAC* address, IP address, and type (actuator or a sensor). This information is shipped to the clustering module for node management.
  (b) Clustering Module: It has information on all connected IoMT nodes, updated by CHs on a requirement basis. For instance, if any node leaves out or a new node/IoMT device joins in the IoMT layer, the CH instantly informs the topology lookup module. This module updates the clustering module, and the clusters are revised.
  (c) Trust Calculator Module: It collects trust-related data (i.e., trust parameters) from the clustering module. It runs the fuzzy logic based on collected trust parameters to assess the trustworthiness of all IoMT nodes. It calculates the trust value based on these parameters. These values lie between 0 and 1, such as 0.3, 0.6, or 0.8; therefore, there is no need to normalize trust values. It also decides whether the node is a Sybil or a normal node, sends the details to the blockchain for storage, and propagates that to the IoMT layer. The underlying devices do not communicate with the malicious ones. The malicious node is isolated from the network. The details of trust calculation, Sybil node detection, and isolation are as follows:
    - Trust Calculation: The Fuzzy Logic model is a trust reference model for an IoT system. The trust model is based on direct and indirect trust. In the 1960s, Lotfi Zadeh [27] presented the idea of Fuzzy Logic (FL). The fuzzy sets used in fuzzy logic comprise group elements that extend the class label to all set items. It is a variant of a Boolean set where all elements have a degree of membership in the range of 0 to 1 [28], where 0 represents no membership, and 1 represents a membership value for an element [29]. This set may be discrete or continuous in nature [30]. For instance, if someone has to buy a motorcycle, he expects that there is a set of motorcycles in his neighborhood that he will purchase. Let X be a set of motorcycles. Each motorcycle has a membership value between 0 and 1 that indicates its degree of participation in the fuzzy set X. These membership values are determined by the bike’s model, price, and condition, among other factors. The amount of the motorcycle price is determined by the membership value. FL operates on these fuzzy sets.
      Due to its ability to deal with ambiguous and uncertain data, common in security contexts, fuzzy logic is useful for identifying internal attacks such as Sybil. Fuzzy logic permits the representation of imprecise and ambiguous concepts, which can be useful for identifying Sybil attacks, which are difficult to detect using conventional methods [31]. By establishing rules that permit considering multiple factors, fuzzy logic can also capture the complex relationships between various variables and their impact on the probability of an attack. It is beneficial for identifying Sybil assaults, which frequently involve multiple identities. It is essential to note, however, that the efficacy of fuzzy logic in identifying Sybil attacks and other security threats is contingent on the quality of the data and the defined rules [5]. Therefore, to determine the true potential of fuzzy logic in detecting Sybil attacks, this paper tests fuzzy logic-based systems on Sybil and non-Sybil data to ascertain their accuracy and efficacy in separating the two.
      The foundation of fuzzy logic is human thinking, taking various values, including True/False, 0/1, Good/Bad, and On/off [32]. It can substantially affect the values falling between false and true, such as partly false and partly true [5]. The values might be anything from false to almost false or half false [1]. It enables machines to think like humans, which helps make judgments in situations similar to actual situations. Its most basic form may be seen as a mapping between the inputs and corresponding outputs of a fuzzy system [33]. This mapping forms a scalar for the output and a vector for the input values. In real-world situations, it may, under certain conditions, help in the decision-making process, facilitating choices that would otherwise be difficult to make using conventional theory. For instance, dealing with road congestion in traffic management is pretty challenging. It is now much simpler to align traffic through input/output mapping, such as traffic flow, road quality, and meteorological conditions [34,35].
  (d) Trust Value Storage on Blockchain and Sybil Node Isolation: The role of blockchain is based on our previous work presented in [15]. Blockchain brings scalability to the system as IoMT devices are diverse and increasing. A network of numerous IoMT devices can be managed securely with blockchain. Also, it handles the secure entries regarding trust values and parameters efficiently. It also behaves as a secure database for the proposed model and helps to detect repeated and duplicated identities of different nodes. Due to all these characteristics, it is used in our model. The CH registration is made using Algorithm 1 [15], IoMT nodes’ trust value is inserted and updated using Algorithm 3 [15], Nodes’ trust verification is made using Algorithm 4 [15], and Sybil node(s) isolation is made using Algorithm 5 [15].
  (e) Trust Value Propagation: Trust value is propagated to CHs for further usage. Whenever an IoMT device wants to communicate, the trust is checked, and the communication is made if it is trustworthy.

### 3.2. Workflow of BFT-IoMT

**Figure 3:**
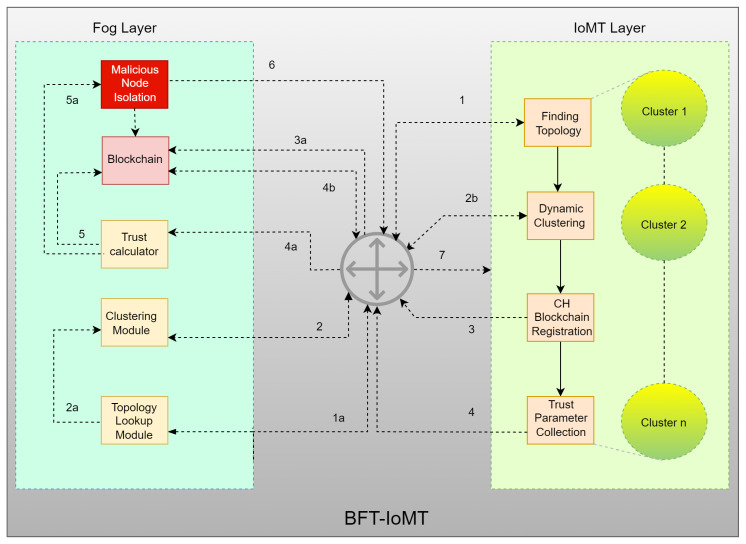
Proposed Architecture.

Figure 3 represents a logical view of the overall functionality and workflow of the proposed BFT-IoMT. The steps are listed below.

1. At initiation, the topology is found using the topology lookup module (Step 1a). This step works whenever an IoMT device/node joins or leaves the underlying IoMT network.
2. In the second step (i.e., 2, 2a, 2b), the clusters and CHs are made based on the knowledge provided in the previous step.
3. Once the clusters and CHs are made, the next step is registering CHs with the blockchain.
4. Now, the trust-related details (i.e., trust parameters) are collected from each IoMT node and sent to the trust calculator module.
5. Once the trust of each IoMT node has been calculated, it is sent to the blockchain for storage. If the assessed trust value is less than the set threshold, it is detected as a malicious (i.e., Sybil) node.
6. Once a Sybil node is detected, it is time to isolate it from the IoMT network.
7. The decision (whether the node is Sybil or benign) is propagated in the whole network.

Algorithm 1 calculates trust, detects and isolates a Sybil node. First of all, the parameters required for the calculations are gathered. In the first step, trust parameters, *MAC* address (MAC), and Residual energy (E_n) of the neighbors in the cluster are collected by each node. E_n is initiated with the value of 100 J for this proposed methodology, and MAC is each node’s unique address. In a Sybil attack, a node makes the identity of one of the neighbor nodes and declares itself as a legitimate node. Therefore, to detect the attack, MAC is monitored after every short interval *d*. Furthermore, this value is stored in a variable named *P*. At this step, this value is checked in the fuzzy set (FS) whether it lies in this set or not. If this condition is satisfied, *P* is considered Trust Value Initial (TV_i). In the next step, the energy of the node (i.e., E_n) is noted to determine whether it is sufficient or not. If E_n of a node is at a sufficient level, then its Trust Value Final (TV_f) is calculated. TV_f is the sum of the Trust Value Sample (TS) and TV_i. After checking both conditions (i.e., steps 3 and 4 in the algorithm), Trust Value Final (TV_f) and TV_i are updated and stored in the blockchain BC. After time *d*, this process is repeated and rechecked to determine the credibility of the nodes. Detecting a malicious or legitimate node is done by comparing the trust value and residual energy parameter values. In step 5 of the Algorithm, again, the condition is checked for Trust Value New and E_n. The new trust value must be greater than the threshold TH, set as 0.5, and E_n must be above 50 J. Both conditions must be true for a legitimate node. A node is marked as malicious if it is below the set threshold (i.e., 50 J). It is isolated and this information is updated in the BC. The decision is shared and propagated in the network in the last step. It is updated in BC and propagated in the network if it is not malicious. In this way, this algorithm not only detects the Sybil attack with the help of blockchain, but also removes the attacker nodes from the network. The proposed algorithm has the run-time value of n number of nodes; thus, its time complexity is O(n). Regarding space complexity, the algorithm takes space for *n* nodes and the number of times due to regular updation.
**Algorithm 1:** Detection and isolation of the Sybil attack.
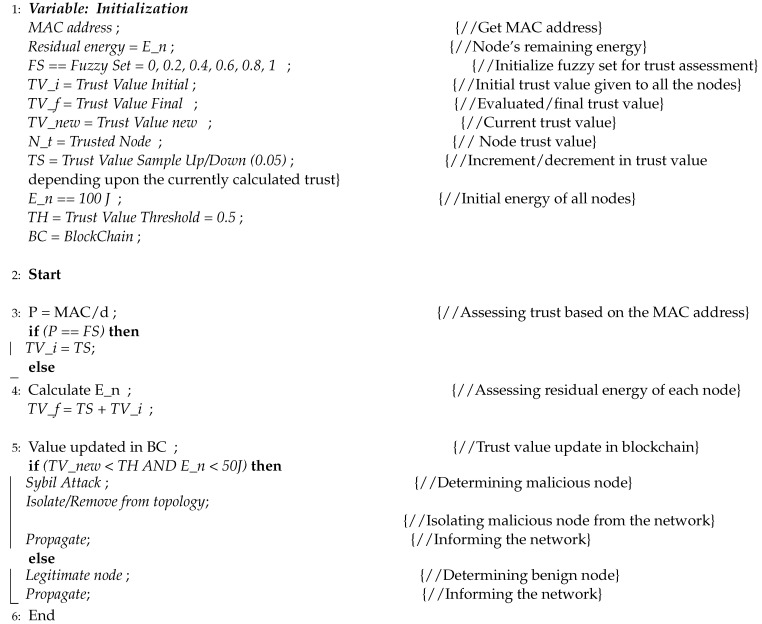


## 4. Experimental Setup and Evaluation

This section details the experimental setup and findings regarding IoMT network lifetime, average residual energy, message overhead, and end-to-end delay.

### 4.1. Experimental Setup

To assess the performance of BFT-IoMT, a series of extensive simulation-based experiments using the Cooja Network Simulator 2.7 is carried out. The network area under consideration is a 100 m × 100 m network region. It is managed with an average speed of 250 Kbps; the radio model reproduces the CC2420 protocol using the default configuration-based 802.15.4 MAC/PHY protocol in the 2.4 GHz band. Every hub (30 hubs were used in total) has a transmission rate of 30 m per second. The IoMT network has 30–120 IoMT nodes, increasing chronologically. The simulation is run on 30 IoMT nodes first for 10, 20, 30, 40, 50, and 60 min (i.e., each simulation is repeated five times). Then the experimentation is made for 60 nodes for the exact simulation times. Later, it is run for 90 and 120 IoMT nodes. In the same fashion, Sybil nodes are introduced. We randomly place 1 to 3 malicious nodes around the IoMT network in each iteration. Malevolent DODAG (Destination Oriented Directed Acyclic Graph [36]) Information Solicitation (DIS) packets are broadcast with 0.1 to 3.0 packets per second Sybil attack rate, where the number of DIS messages represent the Sybil attack rate having fictitious IDs at any given time. The assessment of BFT-IoMT is based on the isolation delay, detection rate, and energy usage. Table 1 contains all the information about the simulation details. At the same time, the details of blockchain tools are summarized in Table 3 in [15].

**Figure 4:**
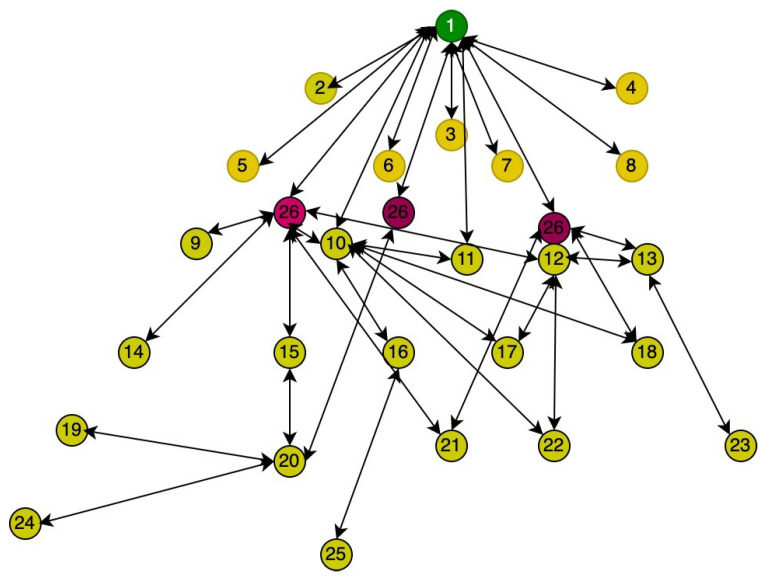
Screenshot of Topology in Cooja.

Figure 4 shows the topology in Cooja during simulation. In the figure, node 26 represents the Sybil attack node having the same identity. The green node represents the border router or sink node. All yellow-colored nodes are legitimate nodes. All these nodes send data to the sink node, and the sink node forwards it to the fog layer.

### 4.2. Results and Discussion

The results of BFT-IoMT are compared with two state-of-the-art schemes presented in [5] called “FTM-IoMT” and [20] called “BlockTrust-RPL”. The comparison is made regarding energy consumption, attack detection, trust computation, packet delivery ratio, and throughput. The details are discussed in subsequent subsections.

#### 4.2.1. Energy Comparison

**Figure 5:**
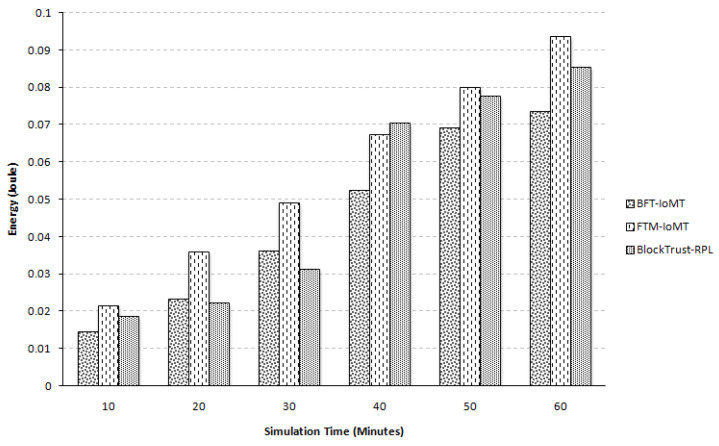
Energy Comparison.

In an IoMT network, many nodes interact with one another to forward data for further assessment in a multi-hop fashion. It uses the energy of the involved node in sending, receiving, and forwarding a data packet. It is to be noted that the energy of a node is also consumed in packet re-sending (if a Sybil attacker drops the data packet). In addition, more energy is consumed in exchanging the trust parameter in trust-based security. Moreover, the energy is used if trust is calculated at the node level. Therefore, it is necessary to conserve the energy at the node level for increased network lifetime. Figure 5 represents the energy comparison between the two frameworks. Cooja network simulators have tools that can provide some statistics about nodes. Energy consumption is also calculated by using one of those tools. The amount of energy consumed is expressed in joules. On average, there is 25.43% more residual energy in the case of BFT-IoMT compared to [5] and for [20], it is 12.64% more energy saving. This percentage is taken for all five iterations with all six simulation times. Due to Trickle Timer Optimization in [20], it produces slightly better results than [5]. This paper also used distributed authentication scheme that helps it to detect it timely. The proposed framework forwards trust assessments and major decision-making to the fog layer. Instead of carrying out trust-related complex computations and decision-making at the node level, they are shipped to the fog layer. This way, the proposed framework conserves a node’s energy in computing and analysis. Furthermore, the decision is also made at the fog layer for detecting and isolating Sybil nodes, which saves a substantial amount of energy compared to the state-of-the-art.

#### 4.2.2. Attack Detection

**Figure 6:**
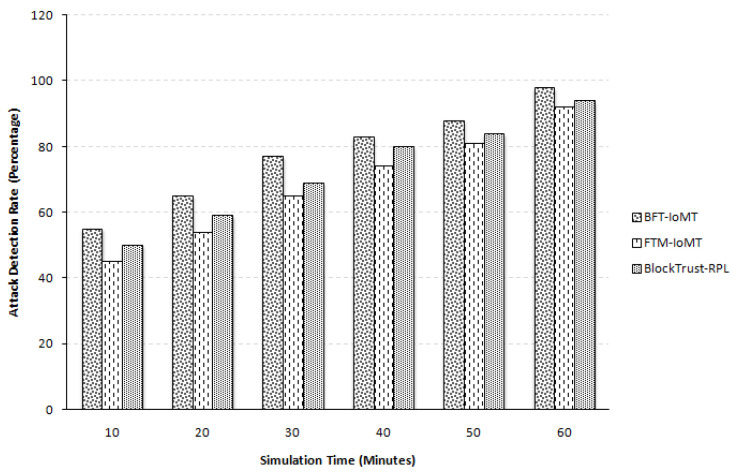
Attack Detection.

Attack detection defines the total number of nodes identified by BFT-IoMT compared to [5,20]. In Figure 6, it can be seen that BFT-IoMT identifies a more significant number of nodes when compared to FTM-IoMT, which is due to the inclusion of fuzzy logic in the evaluation model of the proposed system. In our case, the average accuracy percentage improvement is 12.54% more than that of FTM-IoMT and it is 6.65% more when campared with [20]. In [20], distributed authentication is utilized for better detection. Due to this nature of authentication, it performs better than [5] but does not perform better than the proposed mechanism. This percentage is taken for all five iterations with all six simulation times. This assessment demonstrates that the BFT-IoMT model is more fault-tolerant than the two.

#### 4.2.3. Trust Computation Reliability and Efficacy

**Figure 7:**
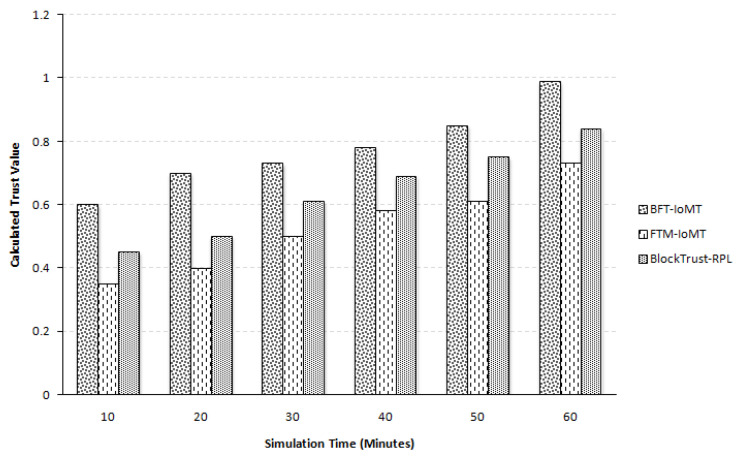
Trust Computations of IoMT Nodes.

The reliability and efficacy of BFT-IoMT, determined in this assessment, are also measured regarding time spent, calculating trust, determining Sybil nodes, and isolating the malicious node. It uses a time setup of 60 min to evaluate nodes’ trustworthiness. Compared to [5,20], the proposed system performs better in the calculation of trust value on an average percentage difference of 37.85% and 19.08%, respectively, as seen in Figure 7. The paper [20] uses valid trust-based parent selection, enabling the mechanism to compute trust values better. These trust values are much more reliable and efficient than the others. Therefore, Ref. [20] performs better than [5]. This percentage is taken for all five iterations with all six simulation times. The proposed framework divides the IoMT layer into clusters, and the CH is responsible for forwarding the trust parameters to the fog layer. The probability of fake/fabricated trust values is also restricted due to blockchain. Furthermore, as mentioned above, it consumes less energy while executing computations than the state-of-the-art.

#### 4.2.4. Packet Delivery Ratio

**Figure 8:**
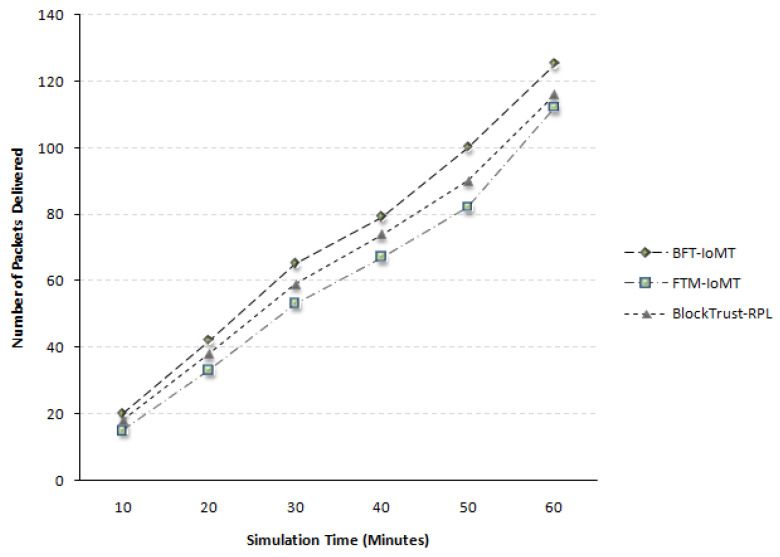
Packet Delivery Ratio.

Figure 8 illustrates the packet delivery ratio in Time(t). The results show that the proposed BFT-IoMT produces a better packet delivery ratio. The packet delivery ratio is the ratio between packets sent from the source and packets received at the destination. It can be calculated by dividing received packets by the total number of packets sent by the source. The results demonstrate a rise of 17.40% more packets delivery compared to [5] and it is 8.72% more compared to [20]. In [20], the concept of Trickle Timer Optimization has been used, which is not available in [5]; therefore, it outperforms the technique used in [5]. Paper [20] also uses distributed authentication, and the method by which it selects the parent node is much better than the selection method of [5]. This percentage is taken for all five iterations with all six simulation times. It is so because the proposed approach uses a fuzzy logic-based trust assessment that is more flexible, allowing moderation among variables in real-time. Secondly, fake nodes cannot propagate fabricated trust values since the calculated trust is saved and referred from the blockchain. This way, the malicious nodes are isolated to ensure successful packet delivery.

#### 4.2.5. Throughput of BFT-IoMT

**Figure 9:**
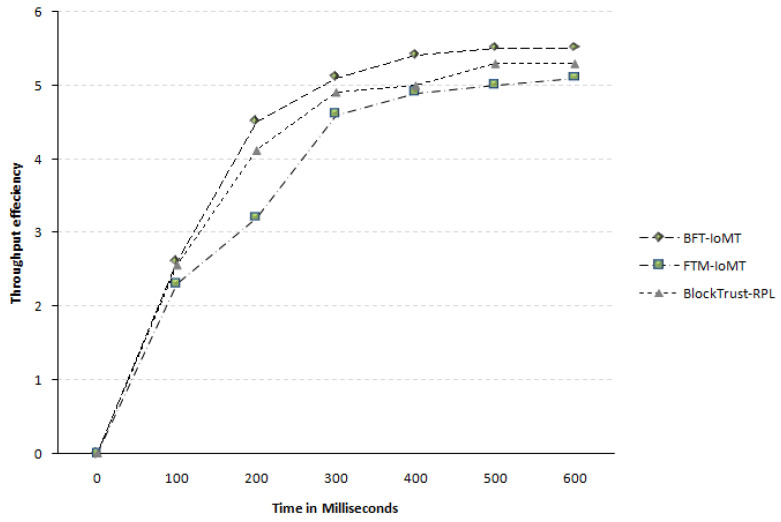
Throughput Efficiency.

The throughput of BFT-IoMT is calculated by counting the number of packets received successfully on the destination node. It measures how well the proposed system performs relative to another scheme’s performance in terms of throughput. Figure 9 illustrates how reliably BFT-IoMT transports data across nodes when compared to the state-of-the-art in terms of time accuracy (t). The results demonstrate a 13.04% and 5.05% more overall throughput efficiency compared to [5,20], respectively. In [20], the concept of Trickle Timer Optimization is used, which is not available in [5] (as mentioned earlier); therefore, it outperforms [5] in terms of throughput. This percentage is taken for all five iterations with all six simulation times. The proposed model outperforms the other approach shown in the graph regarding throughput efficiency. It is so because the proposed BFT-IoMT framework is also based on fuzzy logic. It is one the best options for training intelligent systems and autonomous decision-making in smart applications. Secondly, we divided the network into clusters, where CHs are responsible for data gathering and forwarding to the fog layer. Thirdly, Sybil nodes are detected and isolated from the network, and therefore with timely isolation, the throughput is improved with an increased delivery ratio.

## 5. Conclusions

In the IoMT domain, several intelligent health monitoring devices communicate and forward data further for analysis and immediate decision-making. In this context, secure communication among health monitoring devices is critical for the timely analysis and decision-making of patient data. Using blockchain technology to facilitate distributed trust mechanisms is a promising method for preventing internal attacks such as Sybil attack, a prevalent issue in RPL-based IoT networks. In this regard, efficient and seamless communication is essential. Therefore, trust-based security provides a reliable and trustworthy environment against internal attacks like Sybil. However, managing reliable communications among IoMT devices is a time-taking and extensive energy-consuming process in a large network. BFT-IoMT, a fuzzy logic- and blockchain-based trust management framework, was proposed in this research. In the paper, extensive simulations were conducted to evaluate its effectiveness in detecting and mitigating Sybil attacks. BFT-IoMT manages trust in smart healthcare systems and is designed for a distributed architecture in which the fog layer delivers all services. The proposed approach detects and isolates Sybil nodes using fuzzy logic for calculating trust values. When a node asks for services, the blockchain reviews the request using stored trust values and allows/stops communication. Extensive simulations are conducted with multiple rounds to authenticate the results. The results showed significant improvements in residual energy, attack detection, trust computation reliability and efficacy, packet delivery ratio, and throughput. In our future work, we aim to reduce the network delay by minimizing the computation overhead associated with the fog layer. We also aim to mitigate other internal attacks, such as wormhole, sinkhole, and jamming attacks.

## Figures and Tables

**Table 1:** Parameters used in the simulation.

Parameter	Value
Simulation Tool	Contiki OS-based Cooja 3.0
MAC	CSMA/CA+ MICMAC
Transport Protocol	IPv6
Topology	Random
Node Type	Tmote Sky
Simulation coverage area	100 m × 100 m
Total No. of nodes	30–120
Malicious nodes	3–12
Legitimate to malicious node ratio	1–100
$R_{x}$ ratio	30–100%
$T_{x}$ ratio	100%
$T_{x}$ range	50 m
Interference range	50 m
Traffic type rate	CBR 6 pkt/min
Packet size	46 byte
Routing protocol	RPL
Network Protocol	IP based
Simulation time	−60 min
Link failure model	UDGM with distance

## Data Availability

Data sharing not applicable.
